# A Novel Cleavage Pattern of Complement C5 Induced by *Chlamydia trachomatis* Infection *via* the Chlamydial Protease CPAF

**DOI:** 10.3389/fcimb.2021.732163

**Published:** 2022-01-11

**Authors:** Liang Peng, Jingping Gao, Zihao Hu, Hongbo Zhang, Lingli Tang, Fuyan Wang, Lei Cui, Shanshan Liu, Yujie Zhao, Hong Xu, Xin Su, Xiaojing Feng, Yiyuan Fang, Jianlin Chen

**Affiliations:** ^1^ Reproductive Medicine Center, Department of Obstetrics and Gynecology, The Second Xiangya Hospital, Central South University, Changsha, China; ^2^ Department of Pathology, The Second Xiangya Hospital, Central South University, Changsha, China; ^3^ Department of Laboratory Medicine, The Second Xiangya Hospital, Central South University, Changsha, China; ^4^ Department of Immunology, School of Basic Medical Science, Central South University, Changsha, China; ^5^ Department of Radiology, The Second Xiangya Hospital, Central South University, Changsha, China

**Keywords:** *Chlamydia trachomatis*, infection, complement C5, C5 convertase, CPAF, nafamostat mesylate

## Abstract

Urogenital *Chlamydia trachomatis* infection is one of the most common bacterial sexually transmitted diseases globally. Untreated *C. trachomatis* infections can ascend to the upper genital tract and establish a series of severe complications. Previous studies using C3^−/−^ and C5^−/−^ mice models demonstrated that C3-independent activation of C5 occurred during *C. trachomatis* infection. However, the mechanism of how chlamydial infection activates C5 in the absence of C3 has yet to be elucidated. To delineate interactions between C5 and chlamydial infection, cleavage products in a co-incubation system containing purified human C5 and *C. trachomatis*-HeLa229 cell lysates were analyzed, and a novel cleavage pattern of C5 activation induced by *C. trachomatis* infection was identified. C5 was cleaved efficiently at the previously unidentified site K970, but was cleaved poorly at site R751. C5b was modified to C5b_Ct_, which later formed C5b_Ct_-9, which had enhanced lytic ability compared with C5b-9. The chlamydial serine protease CPAF contributed to C3-independent C5 activation during *C. trachomatis* infection. Nafamostat mesylate, a serine protease inhibitor with a good safety profile, had a strong inhibitory effect on C5 activation induced by chlamydial infection. These discoveries reveal the mechanism of C3-independent C5 activation induced by chlamydial infection, and furthermore provide a potential therapeutic target and drug for preventing tubal fibrosis caused by chlamydial infection.

## 1 Introduction

Rapid growth of sexually transmitted diseases (STDs) worldwide in the 21^st^ century has caused many serious public health problems (Levy et al., 2019). Urogenital *Chlamydia trachomatis* infection is one of the most common STDs globally, with approximately 127.2 million new cases each year (Sieving et al., 2019), and its symptoms are relatively insidious. Approximately 80% of infected females and up to 50% of infected males are asymptomatic (Stamm, 1999; Malhotra et al., 2013), which reduces awareness of the need for infected individuals to seek medical treatment (Bianchi et al., 2016). Untreated *C. trachomatis* infections can ascend to the upper genital tract and establish a chronic infection, potentially leading to serious complications including ectopic pregnancy, pelvic inflammation disease, and tubal factor infertility (den Heijer et al., 2019; Hoenderboom et al., 2019; Reekie et al., 2019). Urogenital *C. trachomatis* infection is therefore often referred to as the “silent killer” of reproductive health.

Fibrosis and occlusion of the fallopian tube, as the pathological basis of tubal factor infertility, are the final stage of the pathological progression induced by *C. trachomatis* infection in the female upper reproductive tract (Growe et al., 2013). Various chlamydial virulence factors, such as chlamydial heat shock protein 60 (cHSP60), outer membrane complex B (OmcB), chlamydial protease like activity factor (CPAF), chlamydial high temperature requirement protein A (CtHtrA), plasmid gene protein (Pgp) 3, Pgp4, and Pgp5, have been demonstrated to be involved in the occurrence of tubal fibrosis (Rodgers et al., 2011; Liu et al., 2014; Huang et al., 2015; Yang et al., 2020). Additionally, many host factors, pathways, and genetic conditions also play critical roles in tubal pathologies during chlamydial infection (Ault et al., 2002; Ramsey et al., 2005; Ohman et al., 2009; Refaat et al., 2009; Hafner, 2015; Yu et al., 2019). Thus, the development of tubal fibrosis induced by chlamydial infection is multifactorial; however, there is no complete theory to fully elaborate the pathological mechanism.

The complement system is a tightly regulated network of proteins that is a critical element of the innate immune response against a wide range of invading microorganisms and can be activated through three pathways: the classical, the mannan-binding-lectin, and the alternative pathways (Sarma and Ward, 2011). These three pathways of complement activation converge at complement component C3. Upon activation of the pathways, C3 is cleaved into C3a and C3b followed by formation of C5 convertases, C4b2a3b or C3bBb3b. C5 convertases hydrolyze C5 at arginine 751 (R751) in the α-chain of C5, releasing a small amino-terminal fragment C5a and exposing the larger fragment, C5b. C5a, as an anaphylatoxin, can promote inflammation responses by binding to C5a receptor. C5b binds four terminal complement components, C6 to C9, leading to the formation of the lytic membrane attack complex (MAC; C5b-9), which disturbs the integrity of cell membranes and induces lysis of cells and microbes (Rus et al., 2005).

The presence of C3 was believed to be indispensable for the assembly of the C5 convertases to generate C5a. However, in addition to the above three pathways, a non-traditional, C3-independent C5 activation pathway has been identified (Huber-Lang et al., 2006). A series of C3-independent C5 convertases such as thrombin, trypsin, kallikrein, plasmin, and factor XIIa can generate complement activation products (Wetsel and Kolb, 1983; Huber-Lang et al., 2006; Krisinger et al., 2012). The cleavage mechanism and products of some of these C3-independent C5 convertases are different from those of C4b2a3b and C3bBb3b (Wetsel and Kolb, 1983; Krisinger et al., 2012). Studies using C3^−/−^ and C5^−/−^ mice models have demonstrated that C3-independent C5 activation occurs in some diseases like acute lung injury (Huber-Lang et al., 2006), LPS-induced fetal loss (Yu et al., 2008), and autoantibody-meditated arthritis (Auger et al., 2012).

In our previous study, 11 inbred strains of mice were compared for their susceptibility to hydrosalpinx (the blockage and filling of a fallopian tube with fluid) induced by *Chlamydia muridarum* (*C. muridarum*) infection. A/J mice, a strain of mice defective in C5, resisted hydrosalpinx after chlamydial infection (Chen et al., 2014). Yang et al. (2014) subsequently reported that C5^−/−^ mice failed to develop any hydrosalpinx following intravaginal infection with *C. muridarum* and displayed significantly reduced inflammatory infiltration and cytokine production in oviduct tissue compared with C5^+/+^ mice. This demonstrated that C5 was required for tubal fibrosis induced by chlamydial infection and may contribute to the pathology by enhancing inflammatory responses. Deficiency in C3 did not affect mouse susceptibility to hydrosalpinx after chlamydial infection (Yang et al., 2014), suggesting that a C3-independent C5 activation pathway occurs in tubal fibrosis induced by chlamydial infection. However, it is not known how chlamydial infection activates C5 in the absence of C3.

The current study aimed to explore the mechanism of C3-independent C5 activation induced by chlamydial infection. The results demonstrate a new pattern of C5 activation, and previously unidentified C5 products were induced by chlamydial infection attributed to CPAF, a chlamydial secreted serine protease. Furthermore, nafamostat mesylate (NM), a serine protease inhibitor with a good safety profile in clinical use, had a strong inhibitory effect on C5 activation induced by chlamydial infection.

## 2 Materials and Methods

### 2.1 Cell Culture and Chlamydial Organisms

HeLa229 cells were purchased from the American Type Culture Collection (ATCC; Manassas, VA, USA) and cultured in Dulbecco’s Modified Eagle’s Medium (DMEM; HyClone, Logan, UT, USA) supplemented with 10% heat-inactivated fetal bovine serum (FBS; Gibco, Grand Island, NY, USA) at 37°C with 5% CO_2_. *C. trachomatis* L2 (L2/434/Bu) was purchased from the ATCC, and a *C. trachomatis* L2 CPAF-deficient strain was kindly provided by Dr. Guangming Zhong (University of Texas Health Science Center, San Antonio, TX, USA), which originated by Dr. Raphael Valdivia (Duke University, Durham, NC, USA) (Snavely et al., 2014). The *C. trachomatis* strains were propagated in HeLa229 cells and purified as described previously (Caldwell et al., 1981). Aliquots of purified chlamydial elementary bodies (EBs) were stored in sucrose-phosphate-glutamate (SPG) storage buffer (220 mM sucrose, 12.5 mM phosphate, and 4 mM l-glutamic acid, pH 7.2-7.4) at −80°C until use.

### 2.2 Preparation of *C. trachomatis*-HeLa229 Cell Lysates

HeLa229 cells were seeded in 6-well plates at a density of 6×10^5^ cells per well and were cultured overnight prior to chlamydial inoculation. After the medium was removed, *C. trachomatis*, diluted in 250 μL SPG, was directly inoculated onto the cell monolayers at an appropriate multiplicity of infection (MOI) as indicated for individual experiments for 2 h at 37°C with 5% CO_2_. An equal amount of SPG without *C. trachomatis* was used to mock infect HeLa229 cells as a control. During chlamydial inoculation, 6-well plates were rocked slowly every 30 min to ensure the inoculum fully contacted with the cell monolayers. Following infection, the inoculum was removed and replaced with DMEM supplemented with 10% FBS. In the penicillin inhibition experiment, 100 U/mL penicillin G (Solarbio, Beijing, China) was added to the medium at the same time. Cells were cultured at 37°C in a 5% CO_2_ incubator. At 0, 12, 24, 36 and 44 h post-infection, cells were washed twice with 1× phosphate-buffed saline (PBS), scraped off the plate, and then suspended in 250 μL PBS per well. The cell suspensions were transferred into 1.5-mL Eppendorf tubes, vortexed with 3-mm glass beads for 1 min, and then centrifuged at 500 g for 10 min at 4°C. The supernatants were collected and used in C5 cleavage assays and hemolysis assays.

### 2.3 *In Vitro* C5 Cleavage Assays

Reactions were carried out at 37°C with a reaction volume of 30 μL in a 1.5-mL Eppendorf tube containing 400 nM purified human C5 (Complement Technologies, Tyler, TX, USA) mixed with 25 μL *C. trachomatis*-HeLa229 cell lysates (*C. trachomatis* infection group), mock-infected HeLa229 cell lysates (mock infection group), PBS (C5 control group) or EBs. In the inhibition assay, *C. trachomatis*-HeLa229 cell lysates were pre-mixed with 400 μM nafamostat mesylate (Abcam, Cambridge, MA, USA) for 10 min, or with 500 μM lactacystin (Glpbio, Montclair, CA, USA) for 30 min respectively, both at 37°C. Reactions were terminated at various time points as indicated for the individual experiments by immediately placed on ice. Cleavage products were subjected to Western blot analysis, enzyme-linked immunosorbent assay (ELISA), and C-terminal sequencing based on liquid chromatography-tandem mass spectrometry (LC-MS/MS).

### 2.4 Western Blot Analysis

Cleavage products (28 μL) were mixed with 7 μL 5× loading buffer (Beyotime Biotechnology, Shanghai, China) and boiled at 95°C for 5 min. Next, 10 μL samples were resolved by SDS-polyacrylamide gel electrophoresis (SDS-PAGE), transferred to polyvinylidene difluoride (PVDF) membranes (Merck Millipore, Billerica, MA, USA), and blocked with 5% BSA in Tris-buffered saline (containing 0.5% Tween 20) for 60 min before incubation with the appropriate antibodies. Primary antibodies used were mouse monoclonal antibody against C5/C5a (Cat# ab11876, Abcam, 1:1000 dilution) to recognize the fragment of C5 containing C5a; mouse monoclonal antibody clones 100a against CPAF (kindly provided by Dr. Guangming Zhong, 1:50 dilution) to recognize the C-terminal 35-kDa fragment of CPAF; and biotin goat polyclonal antibodies against *C. trachomatis* (Cat# ab20387, Abcam, 1:1000 dilution) to recognize the 40-kDa chlamydial major outer membrane protein (MOMP). The bound primary antibodies were incubated with corresponding HRP-conjugated secondary antibodies (Cat# SA00001-0 and SA00001-1, ProteinTech Group, Chicago, IL, USA, 1:5000) and detected through enhanced chemiluminescence by using Luminata Classico Western HRP Substrate (Merck Millipore, Billerica, MA, USA).

### 2.5 ELISA

Quantitative analysis of C5a in the cleavage products were analyzed using a commercially available human C5a ELISA kit (CUSABIO BIOTECH, Wuhan, China) according to the manufacturer’s instructions. A 1:1000 dilution was selected as the optimum dilution ratio of the sample based on the results of pre-experiments.

### 2.6 C-Terminal Sequencing Based on LC/MS-MS Analysis

#### 2.6.1 Sample Preparation

Cleavage products resolved by SDS-PAGE as described in section 2.4 were detected with Coomassie blue staining. Target gel bands were excised, cut into fragments, washed, and dried prior to rehydration with 15 ng/μL trypsin in 50 mM ammonium bicarbonate. After incubation overnight at 37°C, peptides were extracted twice with an extraction solution containing 50% acetonitrile and 5% formic acid, and were lyophilized to near dryness. Part of the sample was then rehydrated with Glu-C in 50 mM ammonium bicarbonate with an overnight incubation at 37°C, followed by extraction and lyophilization as described above. Peptides were resuspended in 10 μL of 0.1% formic acid before LC-MS/MS analysis.

#### 2.6.2 Nano LC

Nanoflow UPLC utilized the Ultimate 3000 system (ThermoFisher Scientific, Waltham, MA, USA) and the nanocolumn was a 150 μm×15 cm in-house made column packed with a reversed-phase ReproSil-Pur C18-AQ resin (1.9 μm, 100 Å, Dr. Maisch GmbH, Germany). The parameters were set as follows: loaded sample volume, 5 μL; mobile phase A, 0.1% formic acid in water; mobile phase B, 0.1% formic acid in acetonitrile; total flow rate, 600 nL/min; LC linear gradient, from 4% to 8% B for 2 min, from 8% to 28% B for 43 min, from 28% to 40% B for 10 min, from 40% to 95% B for 1 min, and from 95% to 95% B for 10 min.

#### 2.6.3 Mass Spectrometry

A Q Exactive™ Hybrid Quadrupole-Orbitrap™ Mass Spectrometer (Thermo Fisher Scientific) was used in the analysis. The parameters were set as follows: spray voltage, 2.2 kV; capillary temperature, 270°C; MS resolution, 70000 at 400 m/z; MS precursor m/z range, 300.0-1800.0; product ion scan range, start from m/z 100; activation type, CID; Min. signal required, 1500.0; isolation width, 3.00; normalized coll. energy, 40.0; default charge state, 6; activation Q, 0.250; activation time, 30.000; data dependent MS/MS, up to top 20 most intense peptide ions from the preview scan in the Orbitrap.

#### 2.6.4 Data Analysis

Raw MS files were analyzed and searched against the sequence of human C5 (NCBI accession number P01031.4) using Byonic. The parameters were set as follows: protein modifications, carbamidomethylation (C) (fixed) and oxidation (M) (variable); enzyme specificity, trypsin or trypsin and Glu-C; maximum missed cleavages, 3; precursor ion mass tolerance, 20 ppm; MS/MS tolerance, 0.02 Da. Only high confident identified peptides were selected for downstream protein identification analysis.

### 2.7 Molecular Dynamics Simulation

The three-dimensional X-ray crystallographic structure of human C5 protein (PBD ID: 3CU7) was obtained from protein data bank (PBD) database. The molecular dynamics simulation of docked complexes was carried out using the Desmond module of Schrödinger software (Schrödinger Release, version 2020). Here, OPLS3e force field was used to initiate the molecular dynamics simulation, and the system was solvated using TIP3 water model. The neutralization of the system was performed by adding counter ions. Energy minimization of the entire system was performed using OPLS3e, as it is an all-atom type force field. The geometry of water molecules, the bond lengths and the bond angles of heavy atoms were restrained using the SHAKE algorithm. Simulation of the continuous system was executed by applying periodic boundary conditions and long-range electrostatics was maintained by the particle mesh Ewald method. The equilibration of the system was performed using NPT ensemble with temperature at 300 k and pressure at 1.0 bar. The coupling of temperature-pressure parameters was done using the Berendsen coupling algorithm. On post-preparation of the system, the production run was performed for 50 ns with a time step of 1.2 fs and trajectory recording was done for every 50 ps summing up to the recording of 1000 frames. The calculation of the Root Mean Square Deviation (RMSD) was conducted for the backbone atoms and was analyzed graphically to understand the nature of protein-ligand interactions.

### 2.8 Hemolysis Assays

The lytic activity of C5b-9 was compared with that of C5b_Ct_-9 by using established chicken erythrocyte lysis assays with modifications (Rawal and Pangburn, 1998; Krisinger et al., 2012). Briefly, to produce C5b,6 or C5b_Ct_,6, 50% normal human serum (NHS; Solarbio, Beijing, China) was incubated with PBS (control group), 10 nM cobra venom factor (CVF; BOYAO, Shanghai, China), *C. trachomatis*-HeLa229 cell lysates, or both *C. trachomatis*-HeLa229 cell lysates and 10 nM CVF for the indicated time at 37°C, respectively. Tubes containing chicken erythrocytes (3×10^7^) in a final volume of 270 μL gelatin veronal-buffered saline with EDTA (GVBE; Complement Technologies) were prepared and kept on ice. An aliquot (30 μL) of the produced C5b,6 or C5b_Ct_,6 was added to the chicken erythrocytes and the mixtures were incubated for 30 min at 37°C. Unlysed cells were removed by centrifugation for 5 min at 3000 g, then the amount of hemoglobin released from the lysed erythrocytes was quantitated by measuring the absorbance of the supernatant at 414 nm. The background signal was established with a control containing 15 μL NHS, 15 μL PBS, and 270 μL GVBE, but no chicken erythrocytes. Chicken erythrocytes incubated with Pierce™ IP Lysis Buffer (Thermo Fisher Scientific) was used as a positive control of 100% lysis.

### 2.9 Proteomic Analysis

RIPA Lysis and Extraction Buffer (Thermo Fisher Scientific) was used for sample lysis and protein extraction. The amount of protein was quantified with the BCA Protein Assay Kit (Bio-Rad, Hercules, CA, USA). Protein digestion by trypsin was performed according to filter-aided sample preparation (FASP) procedure (Wiśniewski et al., 2009). The digest peptides of each sample were desalted on Empore™ SPE Cartridges C18 (Sigma-Aldrich, **S**t. Louis, MO, USA), concentrated by vacuum centrifugation and reconstituted in 40 µl of 0.1% (v/v) formic acid. LC-MS/MS analysis was performed on a Q Exactive mass spectrometer (Thermo Fisher Scientific) that was coupled to Easy nLC (Thermo Fisher Scientific). The MS raw data for each sample were combined and searched using the MaxQuant 1.5.3.17 software for identification and quantitation analysis.

### 2.10 Molecular Docking

The three-dimensional structure of CPAF protein (PBD ID: 3DOR) was obtained from PBD database. The three-dimensional structure of NM was obtained in the PubChem (https://pubchem.ncbi.nlm.nih.gov/). The NM and CPAF structures were processed by PyMOL (version 2.3) and AutoDockTools (version 1.5.6). Molecular docking was performed using Autodock 4 (Morris et al., 2009) to verify the binding activity of NM and CPAF. The final docking structure of the complex was evaluated according to the binding energy. The docking results were visualized in PyMOL.

### 2.11 Statistical Analysis

Data are presented as means ± standard deviation (SD). All data were analyzed with GraphPad Prism 8 (GraphPad Software Inc., La Jolla, CA, USA) using two-tailed unpaired Student’s *t* tests and one-way ANOVA with Dunnett’s multiple comparisons test if three or more groups were compared. P <0.05 was considered statistically significant.

## 3 Results

### 3.1 *C. trachomatis* Infection Induces Unique Undescribed C5 Cleavage Products Independent of C3

**Figure 1:**
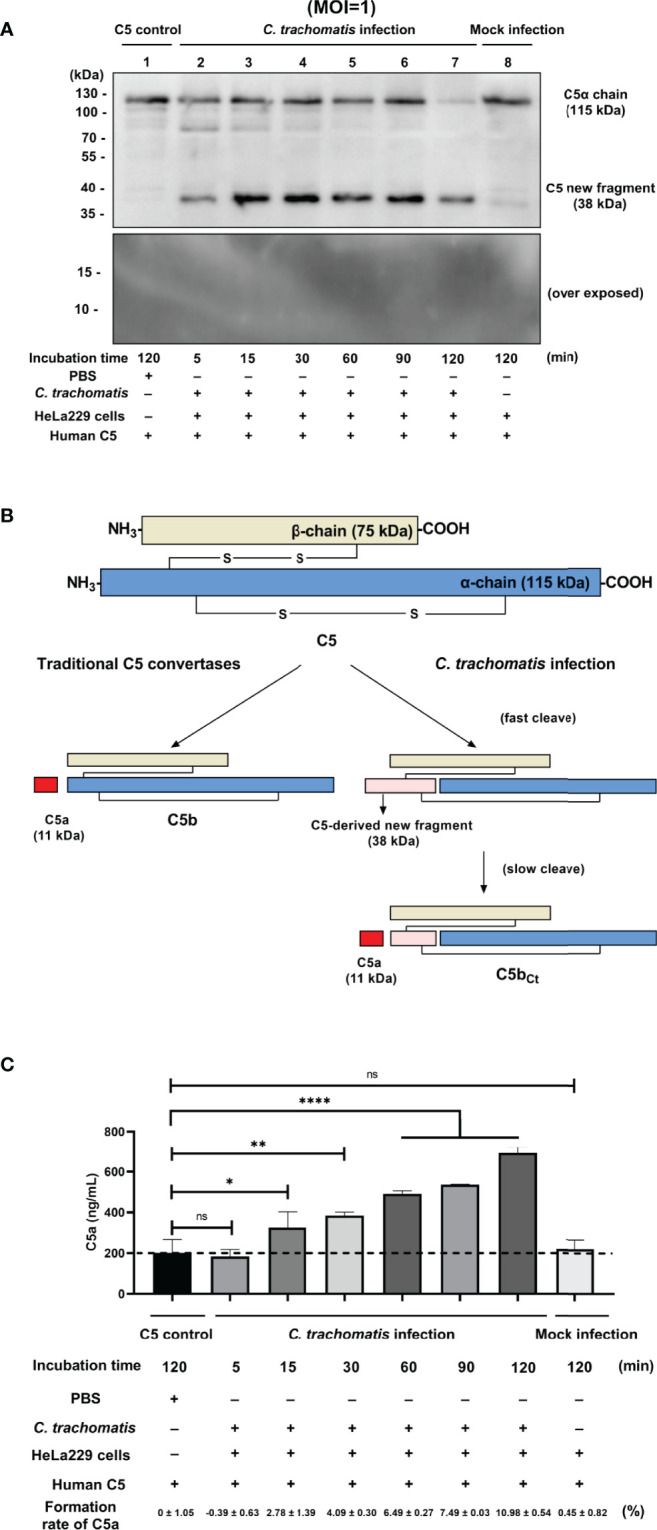
*C. trachomatis* infection yielded a novel undescribed C5 fragment independent of C3. **(A)** *C*. *trachomatis*-HeLa229 cell lysates (MOI=1) incubated with human purified C5 (400 nM) produced a previously undescribed ~38-kDa fragment encompassing C5a in Western blot analysis. **(B)** Schematic overview of proteolytical processing of C5 induced by traditional C5 convertases or *C*. *trachomatis* infection. **(C)** *C*. *trachomatis* infection generated a limited amount of C5a in the absence of C3 detected by ELISA. The C5 control group was taken as the baseline. Formation rates of C5a were calculated according to the concentration of human C5 protein before the reaction (400 nM). ns, not significant; *P < 0.05; **P < 0.01; ****P < 0.0001 (Dunnett’s multiple comparisons test, *n*=3).

To explore the mechanism of C3-independent C5 activation induced by *C. trachomatis* infection, a co-incubation system containing purified human C5 (400 nM) and *C. trachomatis*-HeLa229 cell lysates (MOI=1) or mock infected HeLa229 cell lysates (mock-infected control) or PBS (C5 control) was established. This system mimics the process of contact between the contents of lysed epithelial cells and C5 in body fluids during genital *C. trachomatis* infection in the absence of C3. An anti-C5a antibody was utilized to detect the fragments of C5 cleavage products encompassing C5a. After incubation for 5 to 120 min, Western blot analysis revealed that co-incubation of C5 and *C. trachomatis*-HeLa229 cell lysates yielded a previously undescribed C5-derived ~38-kDa fragment encompassing C5a (**Figure 1A**, Lines 2-7). This band was not detected in the C5 control (**Figure 1A**, Line 1) or the mock-infected control (**Figure 1A**, Line 8) after incubation for 120 min. Unlike the traditional C5 cleavage pattern (**Figure 1B**), an 11-kDa C5a band was not detected in any of the samples, despite optimization of Western blot conditions for this small molecular weight protein.

To investigate whether the C5a generated in co-incubation system was below the limitation of detection of Western blot analysis, an ELISA was subsequently performed. The ELISA showed that there was a certain amount of C5a in the C5 control group (**Figure 1C**). This could be due to the only 95% purity of the human C5 protein and the possible presence of a small amount of C5a impurities in it. Thus, the concentration of C5a in the C5 control group was taken as the baseline in the following comparison. In the *C. trachomatis* infection group, an increase in C5a content was not detected after incubation for 5 min (P > 0.05). However, after incubation for 15 to 120 min, the concentration of C5a in the *C. trachomatis* infection group significantly increased (15 min: P < 0.05; 30 min: P < 0.01; 60 min, 90 min, and 120 min: P < 0.0001) compared with that of the C5 control group. Moreover, the concentration of C5a gradually increased over the incubation time. In contrast, the concentration of C5a in the mock infection group was not statistically different (P > 0.05) to that of the C5 control group, congruent with Western blot results. This suggested that the process of incubation itself or the presence of HeLa229 cells lysates alone did not produce the C5 cleavage effect, and that cleavage of C5 observed in the *C. trachomatis* infection group was induced by chlamydial infection. The formation rates of C5a were calculated based the concentration of human C5 protein (400 nM) added into the co-incubation system. After 120 min of incubation, the formation rate of C5a was only 10.98 ± 0.54% for the *C. trachomatis* infection group.

### 3.2 The Unique Cleavage Mechanism of C5 Induced by *C. trachomatis* Infection

To identify the amino acid sequence of the previously undescribed C5-derived ~38-kDa fragment encompassing C5a in Western blot analysis, C-terminal sequencing based on LC-MS/MS was performed. Excised bands from SDS-PAGE gels containing the ~38-kDa fragment were digested by trypsin, and half of the obtained peptides were further digested by Glu-C. The two groups of peptides were analyzed by LC-MS/MS and the obtained peptide information was correlated with the amino acid sequence of human C5 (NCBI search number: P01031.4) for comparison. Sequences of peptides identified by trypsin digestion or trypsin & Glu-C digestion are shown in **Table 1**. The peptides closest to the C-terminal were both IPLDLVPK and corresponded to I963-K970 of C5. As the ~38-kDa fragment encompassing C5a (the N-terminal of C5 α-chain) was detected with the anti-C5a antibody, the sequence of the novel ~38-kDa fragment corresponded to T678-K970 of C5.

**Table 1:** Sequences of peptides identified by trypsin digestion or trypsin and Glu-C digestion of the novel C5-derived ~38-kDa fragment.

Digestion method	Sequence	Position in C5 sequence	Number of sequences identified
Trypsin	TLLPVSKPEIR	756-766	2
	GEQIQLK	836-842	1
	VVPEGVK	929-935	1
	VVPEGVKR	929-936	1
	ESYSGVTLDPR	937-947	2
	IPLDLVPK	963-970	2
Trypsin & Glu-C	SYFPE	767-771	1
	MNIPYSVVR	827-835	1
	QIQLK	838-842	1
	VVPEGVK	929-935	1
	ESYSGVTLDPR	937-947	1
	KEFPYR	957-962	1
	IPLDLVPK	963-970	1

**Figure 2:**
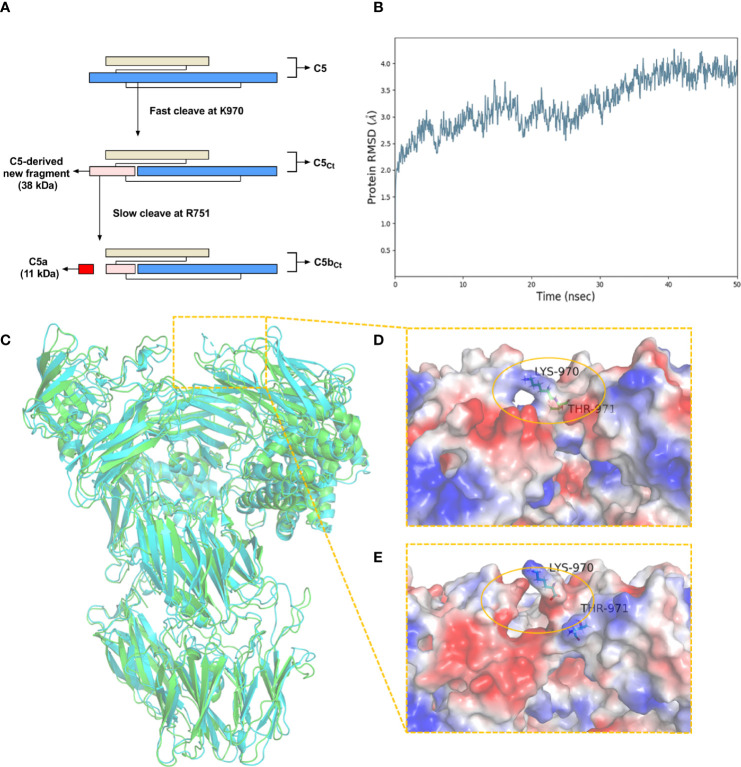
The unique cleavage mechanism of C5 induced by *C*. *trachomatis* infection. **(A)** The cleavage pattern and cleavage sites of C5 induced by *C. trachomatis* infection. **(B)** Plot of RMSD for C5_Ct_ for 50 ns of simulation. **(C)** The superposition of the structure of C5 (green) and C5_Ct_ (cyan). **(D)** A close-up view of the electrostatic surface of C5 at residues 970-971. **(E)** A close-up view of the electrostatic surface of C5_Ct_ after peptide bond breaking between residues 970-971.

By combining the results from Western blot analysis, ELISA, and C-terminal sequencing based on LC/MS-MS, the unique cleavage mechanism of C5 induced by *C. trachomatis* infection could be predicted (**Figure 2A**). In the traditional C5 cleavage pattern, C4b2a3b or C3bBb3b, as C5 convertases, rapidly hydrolyze C5 at R751 in the α-chain of C5 and release C5a and C5b. However, in the distinct C5 cleavage pattern induced by *C. trachomatis* infection, the K970 site was first cleaved efficiently. During physiological conditions, the C5-derived ~38-kDa fragment detected in Western blot analysis and still connected with C5 α-chain and C5 β-chain by disulfide linkages, which resembles C5, is herein referred to as C5_Ct_. The time at which the band corresponding to the C5-derived ~38-kDa fragment was detected by Western blot analysis was earlier than the C5a level elevated in the ELISA, suggesting that cleavage of the R751 site was more slowly induced by *C. trachomatis* infection compared with cleavage of the K970 site. After releasing C5a, C5_Ct_ was modified into a three-chained disulfide-linked molecule that resembled C5b, herein referred to as C5b_Ct._

A molecular dynamics simulation was conducted to predict the conformational change of C5 after the cleavage of the K970 site. The RMSD was stable after 40 ns and converged at 3.8 Å (**Figure 2B**). The superposition of C5 and C5_Ct_ (**Figure 2C**) showed that the overall conformational structure of C5_Ct_ did not undergo a significant change after breakage of the peptide bond between residues 970 and 971, which indicated that C5_Ct_ might be similar to C5 in the process of C5b_Ct_-9 assembly. However, in the locality of peptide-bond cleavage between residues 970 and 971, the electrostatic surface was no longer as smooth as it was before the fracture (**Figures 2D, E**). The next part of the investigation therefore focused on whether this change made a difference to the lytic activity of C5b-9 and C5b_Ct_-9.

### 3.3 C5b_Ct_-9 Modified by *C. trachomatis* Exhibits More Lytic Activity Than C5b-9

**Figure 3:**
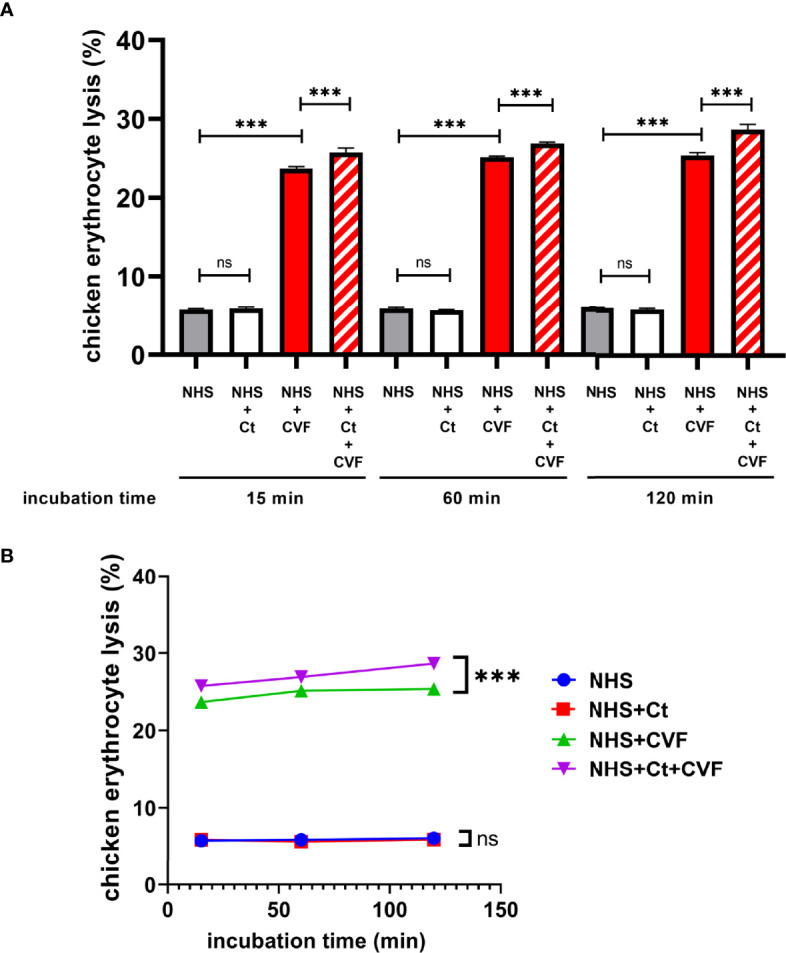
C5b_Ct_-9 modified by *C. trachomatis* exhibits more lytic activity than C5b-9. **(A)** Hemolysis assays showed that incubation of both *C*. *trachomatis*-HeLa229 cell lysates and CVF with NHS significantly enhanced the lytic activity of the resulting products. **(B)** Line chart showing the lysis rate of erythrocytes was almost constant over the incubation time. ns, not significant; ***P < 0.001 (Dunnett’s multiple comparisons test, *n*=3). NHS, Normal human serum; Ct, *C*. *trachomatis*-HeLa229 cells lysates; CVF, Cobra venom factor.

During the genital infection of *Chlamydia*, lysis of infected host cells and excessive tissue damage might be associated with overactivation of the complement system, especially due to the enhanced lytic activity of MAC (i.e., C5b-9 and C5b_Ct_-9). There were two possible causes for the enhanced overall MAC function during *C. trachomatis* infection: 1) *C. trachomatis* infection promoted the generation of MAC, resulting in the total number of MAC increasing. 2) The lytic ability of a single MAC enhanced after the modification from C5b-9 to C5b_Ct_-9, due to structural changes. To verify these hypotheses, NHS was mixed with *C. trachomatis*-HeLa229 cell lysates or/and C5 convertase in a co-incubation system, and the lytic activity of these products was determined by chicken erythrocyte lysis assays (**Figure 3A**).

Firstly, evaluation of whether *C. trachomatis* infection directly increased the amount of MAC was conducted. The product of NHS and PBS incubation served as a control group to verify the background lytic activity of NHS. Compared with the control group, the product of NHS and *C. trachomatis*-HeLa229 cell lysate incubation was not statistically different after 15, 60, and 120 min, respectively (P > 0.05), which suggested that *C. trachomatis* infection failed to generate enough MAC, due to slow cleavage of the R751 site and lack of C5b or C5b_Ct_.

Next, the difference in the lytic ability between C5b-9 and C5b_Ct_-9 was determined. Excessive C5 convertase was used for cleavage at the R751 site that would release enough C5b to assemble C5b-9. Since C5 convertase C3bBb3b has a short half-life (1.5-3 min), CVF was mixed with NHS to assemble CVF,Bb, a more stable C5 convertase that has a longer half-life (~7 h) and similar kinetic properties to C3bBb3b with an indistinguishable Km and k_cat_, for rapid cleavage of C5 at R751 (DiScipio et al., 1983; Rawal and Pangburn, 2000; Krisinger et al., 2012). As predicted, the product of NHS and CVF incubation showed a significantly stronger lytic activity than that of the control group at all incubation times (P < 0.001), and the lysis rate of erythrocytes was almost constant over the incubation period (**Figure 3B**). These findings suggested that CVF,Bb had cleaved most C5 into C5b in a short time and an abundance of C5b-9 was assembled. Incubation of *C. trachomatis*-HeLa229 cell lysates together with CVF and NHS slightly but statistically significantly increased the product lytic activity compared with the product of NHS and CVF incubation (P < 0.001). Taken together, these results revealed that C5b_Ct_-9 exhibited more lytic activity than C5b-9.

### 3.4 Chlamydial Infection After 24 Hours Post-infection But Not EB Cleaves C5 at K970 Site

**Figure 4:**
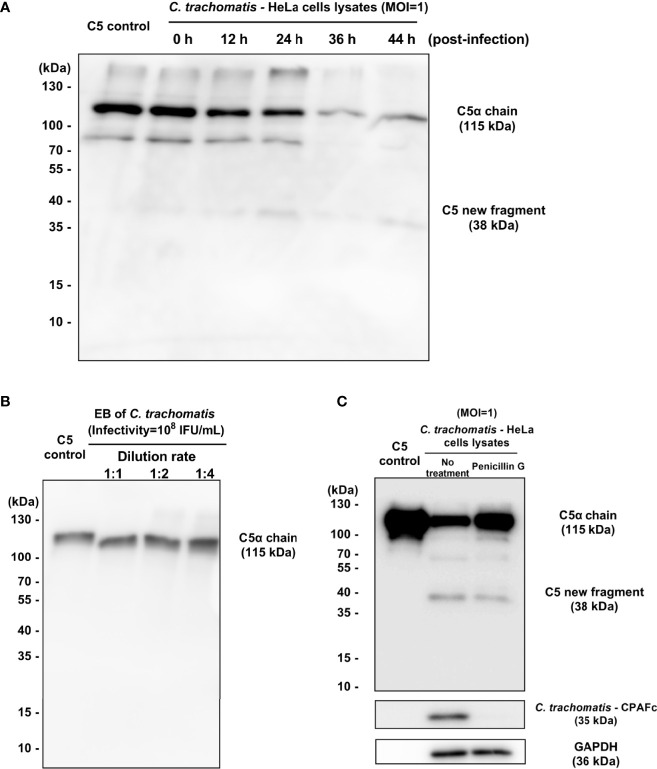
Chlamydial infection after 24 hours post-infection but not EB cleaves C5 at K970 site. **(A)** The **(C)** trachomatis-HeLa229 cell lysates (MOI=1) collected at 0 h, 12 h, 24 h, 36h and 44 h post-infection were co-incubated with purified C5 (400 nM) for 120 min. Western blot analysis showed the fragments encompassing C5a. **(B)** The purified chlamydial EBs were co-incubated with purified C5 (400 nM) for 30 min. Western blot analysis showed the fragments encompassing C5a. **(C)** Penicillin treatment significantly reduced the consumption of the band of C5α chain, and also inhibited the expression of CPAF.

To explore the correlation between the developmental cycle of the Chlamydia and the cleavage of C5, the *C. trachomatis*-HeLa229 cell lysates (MOI=1) collected at 0 h, 12 h, 24 h, 36h and 44 h post-infection were co-incubated with purified C5 (400 nM) for 120 min. The results (**Figure 4A**) showed that the lysates collected at 24 h post-infection yield a faint band of ~38-kDa fragment. The significant reduction of the bands of C5α chain demonstrated that C5 was largely cleaved by the lysates collected at 36 h and 44 h post-infection. Since each developmental cycle of *C. trachomatis* L2 is ~48 h, these results indicated that only the chlamydial infection in the middle and late stage of the developmental cycle had the cleavage effects on C5.

*Chlamydia* has a unique biphasic lifestyle involved in the alternate between two morphologically distinct forms, an infectious non-replicative elementary body (EB), and a replicative, non-infectious reticulate body (RB) (O’Connell and Ferone, 2016). After the invasion into host cells, EBs differentiate into RBs to grow and divide. When replication is completed, RBs convert back to EBs asynchronously in the middle and late stage of the developmental cycle. To determine whether chlamydial EBs was the effective component in the *C. trachomatis*-HeLa229 cell lysates that had directly cleavage activity on C5, purified chlamydial EBs, with the same infectivity of 10^8^ IFU/mL as the *C. trachomatis*-HeLa229 cell lysates (MOI=1) used in previous experiments described above, were co-incubated with purified C5 (400 nM) for 30 min. The result (**Figure 4B**) showed that EB failed to cause the cleavage of C5. Since the purification process of EBs removed host cell components and soluble proteins from lysates, this observation suggested that the unknown C3-independent C5 convertase induced by *C. trachomatis* infection was a chlamydial secretory protein or a newly protein derived from host cells, synthesized in the middle and late stage of chlamydial infection.

In order to further explore the relationship between the middle and late stage of chlamydial infection and the cleavage of C5, a model of penicillin-induced persistence of infection with *C. trachomatis* reported previously (Dille et al., 2014) was used. HeLa229 cells infected with *C. trachomatis* L2 (MOI=1) with or without addition of 100 U/mL Penicillin G. Lysates collected at 44 h post-infection were co-incubated with purified C5 (400 nM) for 120 min. The results (**Figure 4C**) showed that penicillin treatment significantly reduced the consumption of the band of C5α chain. The expression of CPAF was also reduced by penicillin treatment, which was consistent with the result reported by Dille (Dille et al., 2014). In this model, the invasion and stimulation of *Chlamydia* to host cells are mostly retained, but due to environmental stress, RBs convert to aberrant bodies instead of EBs. Thus, the result suggested the middle and late stage of chlamydial infection is closely related to C5 cleavage, and also demonstrated that the invasion of *Chlamydia* didn’t induce host cells to secrete the new protease that cleave C5.

To identify this unknown C3-independent C5 convertase occurring in the middle and late stage of chlamydial infection, a mass spectrometry-based label-free quantitative proteomics analysis of the lysates at 24 h post-infection was conducted. Compared with the mock infection group as a control, there were 57 human proteins and 259 chlamydial proteins only identified in the infection group but not the mock infection group (shown in **Table S1**). No known C5 convertases, such as conventional C5 convertases (C4b2a3b or C3bBb3b), thrombin, trypsin, kallikrein, plasmin, factor XIIa and so on, in the differentially expressed proteins. To our knowledge, all known C5 convertases are serine proteases (Rawal and Pangburn, 2001). According to annotated function in the protein database, three serine proteases in the differentially expressed proteins were identified as candidate C5 convertases: Tripeptidyl-peptidase 1 (TPP1), CPAF and cHtrA. Interestingly, the result also showed that Glia-derived nexin, a serine protease inhibitor derived from host cells, was induced by *C. trachomatis* infection, with inhibitory activity toward thrombin, trypsin, and urokinase that are all C3-independent C5 convertases. It seemed that the host response tended to inhibit rather than upregulate C5 convertase activity during the chlamydial infection.

Among 3 candidate C5 convertases identified by proteomics analysis, CPAF, a chlamydial secreted serine protease, with the potential to cleave or degrade a wide range of host proteins including complement factor C3 and factor B (Zhong, 2009; Yang et al., 2016), showed some correlations with C3-independent C5 activation in the experiments described above. On the one hand, several previous researches reported that although CPAF protein was detected inside inclusions as early as 12 h post-infection, clear CPAF secretion and activity were only detected 24 h after infection (Zhong et al., 2001; Huang et al., 2008). The expression profile of activated CPAF was consistent with the time points during the chlamydial infection that activated C5. On the other hand, Zhong et al. demonstrated that no CPAF or CPAF activity was contained in purified EBs (Zhong et al., 2001) that showed no cleavage effects on C5 in our experiment. Therefore, CPAF was considered the candidate with the highest priority for C3-independent C5 convertase during chlamydial infection.

### 3.5 CPAF Participates in C3-Independent C5 Activation Induced by Chlamydial Infection

**Figure 5:**
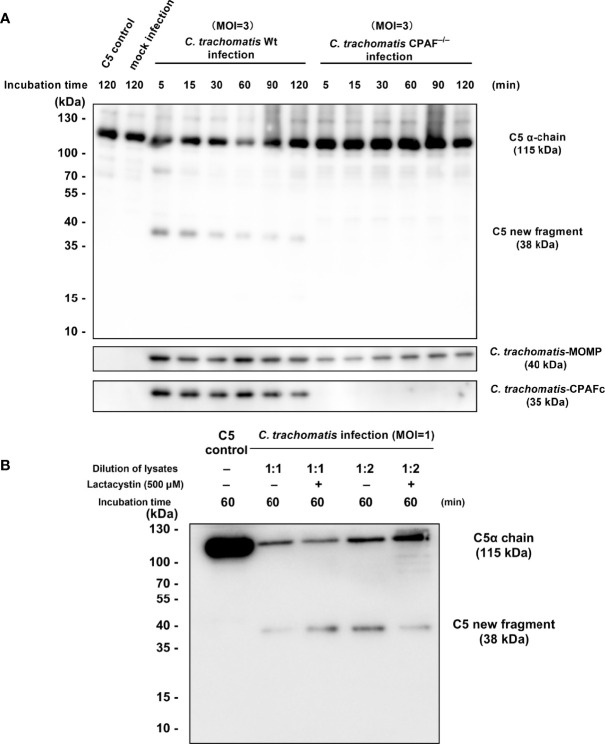
CPAF induces a C5-derived novel fragment independent of C3. **(A)** Purified C5 was incubated with PBS, HeLa229 cells lysate, *C*. *trachomatis*-HeLa229 cells lysate, or *C*. *trachomatis* CPAF^−/−^-HeLa229 cells lysate, respectively. Products of the incubation were subjected to immunoblot analysis against C5a, MOMP, and CPAFc. **(B)** Lactacystin (500 μM) failed to inhibit C5 cleavage induced by 1:1 dilution of *C. trachomatis*-HeLa229 cell lysates, but slightly reduced consumption of the band corresponding to the C5α chain (115 kDa) in the group of 1:2 dilution of *C. trachomatis*-HeLa229 cell lysates.

To further investigate the correlation between CPAF and C5 activation, a *C. trachomatis* L2 CPAF-deficient (CPAF^−/−^) strain was used for the preparation of *C. trachomatis* CPAF^−/−^-HeLa229 cell lysates for use in the co-incubation system for C5 cleavage. The MOI was increased to 3 to ensure an adequate amount of CPAF^−/−^ strain was present in the co-incubation system due to CPAF mutants producing less infectious yield than wild-type *C. trachomatis* (Snavely et al., 2014). Western blot analysis (**Figure 5A**) revealed distinct bands corresponding to the C5-derived ~38-kDa fragment in the wild-type infection group after incubation for 5 and 15 min. The bands gradually became thinner with prolonged incubation time. *C. trachomatis* CPAF^−/−^-HeLa229 cell lysates incubated with C5 did not produce the ~38-kDa fragment after incubation from 5 to 120 min. In both the wild-type group and the CPAF^−/−^ group, bands corresponding to MOMP were detected, which verified that the two co-incubation systems contained adequate amounts of *C. trachomatis.* In the CPAF^−/−^ group, there was no band corresponding to CPAF as expected. This combination of results indicated that CPAF participated in a C3-independent C5 activation during chlamydial infection, but not distinguished between the two possibilities: 1) CPAF directly cleaved C5. 2) An unknown protein derived from host cell upregulated due to CPAF, and then cleaved C5.

Lactacystin, as a CPAF inhibitor reported previously (Zhong et al., 2001; Huang et al., 2008), was pre-mixed with *C. trachomatis*-HeLa229 cell lysates followed by co-incubation with purified C5 protein. Western blot analysis (**Figure 5B**) showed that lactacystin had no significant inhibitory effect on C5 cleavage triggered by 1:1 dilution of *C. trachomatis*-HeLa229 cell lysates, but slightly reduced consumption of the band corresponding to the C5α chain (115 kDa) in the group of 1:2 dilution of *C. trachomatis*-HeLa229 cell lysates.

### 3.6 NM Inhibits the C3-Independent C5 Activation Triggered by Chlamydial Infection and Has a Reasonable Inhibitory Conformation With CPAF

**Figure 6:**
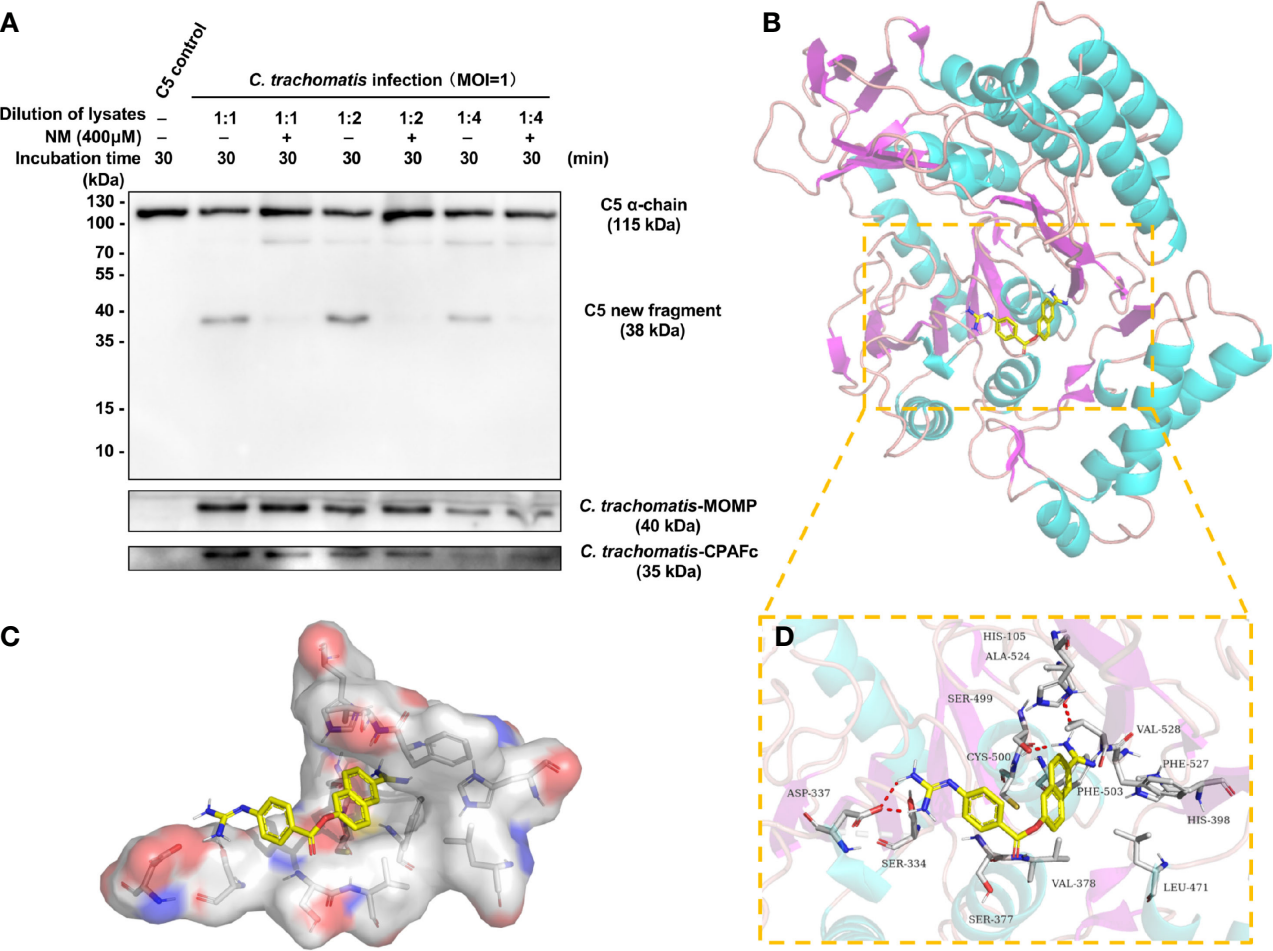
NM inhibits the C3-independent C5 activation triggered by chlamydial infection and has a reasonable inhibitory conformation with CPAF. **(A)** NM (400 μM) was pre-mixed with different dilutions of *C*. *trachomatis*-HeLa229 cell lysates. Purified C5 was then incubated with PBS or the *C*. *trachomatis*-HeLa229 cell lysates for 30 min, respectively. The resulting products were subjected to immunoblot analysis against C5a, MOMP, and CPAFc. **(B)** The 3D structure of NM-CPAF complex. **(C)** The surface of the active site of CPAF. **(D)** The detail binding mode of NM-CPAF complex. The backbone of CPAF was rendered in tube and colored in bright blue. NM was colored in yellow. The hydrogen bonds in NM-CPAF complex were shown in red dashed lines.

NM, a synthetic serine protease inhibitor with a good safety profile, is widely used to inhibit the activity of the complement system (Watkins et al., 1989; Schwertz et al., 2008). To evaluate whether NM could inhibit the C3-independent C5 activation triggered by chlamydial infection, NM (400 μM) was pre-mixed with different dilutions of *C. trachomatis*-HeLa229 cell lysates followed by co-incubation with purified C5 protein. Western blot analysis revealed that NM completely blocked the C3-independent C5 activation triggered by different dilutions of *C. trachomatis*-HeLa229 cell lysates (**Figure 6A**), suggesting that NM had an inhibitory effect on the C5 activation induced by chlamydial infection.

To further explore whether there was a reasonable conformation between NM and CPAF, a molecular docking with NM and CPAF was conducted. Molecular docking programs perform a search algorithm in which the conformation of the ligand is evaluated recursively until the convergence to the minimum energy is reached (Pagadala et al., 2017). The binding energy between NM and CPAF was calculated to predict their affinity. A binding energy lower than 0 indicates that two molecules combine spontaneously and that smaller binding energies lead to more stable conformations. The results showed that the binding energy between NM and CPAF was -7.34 kcal/mol, suggesting a strong and stable binding between NM and CPAF. The NM-CPAF complex obtained from precise docking in Autodock and visualized in PyMOL, unraveling the *in silico* binding inhibitory mechanisms. According to previous research reports, the active site of CPAF is a water-mediated catalytic triad containing His105, Ser499 and Glu558 (Huang et al., 2008). NM specifically bound to the active site of CPAF (**Figures 6B, C**). The hydrophilic parts at both ends of NM formed hydrogen bonds with Ser377, Ser499, and Ala524 (**Figure 6D**), which played important roles in stabilizing the conformation of NM-CPAF complex. A stable occupation of NM in the active site of CPAF near two important catalytic residues, His105 and Ser499 (**Figure 6D**), may explain the inhibition of NM on CPAF. Due to the strong interaction of NM with active site of CPAF, NM might be considered as an effective CPAF inhibitor. Combined with the fact that NM inhibited the cleavage of C5 induced by *C. trachomatis*-HeLa cells lysates, these docking results also enhance the likelihood that CPAF interacts directly with C5.

## 4 Discussion

The complement system is a network of proteins that play a key role in innate and adaptive immunity through the clearance of pathogens and tissue homeostasis (Sarma and Ward, 2011; Mastellos et al., 2019). A C3-independent C5 activation pathway was previously demonstrated to contribute to tubal fibrosis induced by chlamydial infection (Yang et al., 2014), but the mechanism by which chlamydial infection activates C5 in the absence of C3 remains to be elucidated. The current study sought to explore this mechanism of C3-independent C5 activation triggered by *C. trachomatis* infection. A co-incubation system containing purified human C5 and *C. trachomatis*-HeLa229 cell lysates was constructed to simulate the interactions among *C. trachomatis*, host cells, and C5 in the absence of C3. The results propose a novel cleavage pattern of C5 induced by *C. trachomatis* infection that is different from the traditional cleavage pattern of C5 in the complement system.

C5 has usually been thought to be solely cleaved at the R751 site, releasing two functional products, C5a and C5b (Sarma and Ward, 2011; Merle et al., 2015). However, activation of C5 induced by *C. trachomatis* yielded a previously undescribed C5-derived ~38-kDa fragment encompassing C5a, instead of the 11-kDa C5a in Western blot analysis. Subsequent analysis by ELISA revealed the presence of a small amount of C5a in the cleavage products that was below the detection limit of Western blot analysis. Compared with reported data for CVF,Bb (Krisinger et al., 2012), *C. trachomatis*-HeLa229 cell lysates incubated with purified C5 had a relatively low generation rate of C5a, with only ~10% C5a was generated after 120 min incubation. After 5 min of incubation, a prominent band corresponding to the ~38-kDa fragment was detected by immunoblotting (**Figure 1A**), while there was no significant elevation of C5a within same incubation time (**Figure 1C**). This demonstrated that the K970 site was cleaved more efficiently and rapidly than the R751 site during C5 activation induced by *C. trachomatis* infection. This cleavage pattern of C5 is relatively similar to that of thrombin reported by Krisinger et al. (Krisinger et al., 2012), in which thrombin cleaved C5 poorly at R751 but efficiently at R947.

In hemolysis assays, incubation of *C. trachomatis*-HeLa229 cell lysates alone with NHS did not enhance the lytic activity of products, which indicated that C5_Ct_ lacked lytic activity and that *C. trachomatis*-HeLa229 cell lysates alone might have limited ability to generate sufficient C5b or C5b_Ct_ due to the low efficiency of cleavage at the R751 site. The adequate amounts of C5b_Ct_-9 produced in a short time *in vitro* relied on the co-existence of traditional C3-dependent C5 convertase cleaving at the R751 site, which appears to contradict the phenomenon that C3^−/−^ mice have no resistance to hydrosalpinx after chlamydial infection (Yang et al., 2014). Likewise, thrombin also produced very small amounts of C5a when incubated with C5 alone (Krisinger et al., 2012). However, in those diseases mediated by thrombin that have a C3-independent C5 activation, such as acute lung injury (Huber-Lang et al., 2006), LPS-induced fetal loss (Yu et al., 2008), and autoantibody-meditated arthritis (Auger et al., 2012), C3^−/−^ mice still had the same disease susceptibility as wild-type mice. A possible explanation for this is that the long course of diseases *in vivo* cannot fully be simulated with an *in vitro* co-incubation system on account of the relatively short incubation time. Urogenital *C. trachomatis* infection is a lengthy process that can take several weeks or months, even up to 4 years (O’Connell and Ferone, 2016). Since approximately 10% C5a was generated after 120 min incubation of *C. trachomatis*-HeLa229 cell lysates and C5 *in vitro* and continuous synthesis of complement components and CPAF occurs *in vivo*, adequate amounts of C5b_Ct_-9 might be produced during the long process of *C. trachomatis* infection even though C3 was deficient.

Incubation of both *C. trachomatis*-HeLa229 cell lysates and CVF with NHS slightly but statistically significantly enhanced the lytic activity of the resulting products, which seemed to be consistent with the slight changes in the structure of C5b_Ct_. The erythrocyte lysis rate was almost constant after co-incubation for 15 min (**Figure 3B**), which was in accordance with the ability of CVF to rapidly and exhaustively activate complement when CVF encounters human serum (Vogel et al., 2004). The quantities of C5b_Ct_-9 and C5b-9 in the two groups with added CVF (*C. trachomatis*-HeLa229 cell lysates + CVF + NHS and CVF + NHS) were almost equal. The difference in lytic ability of products between these two groups was attributed to the difference in lytic ability between C5b_Ct_-9 and C5b-9, rather than the difference in quantity of them. Thus, the data proved that C5b_Ct_-9 exhibited more lytic activity than C5b-9. The association between C5b-9 and fibrosis has been reported in several fibrotic diseases like idiopathic rapidly progressive glomerulonephritis (Gionanlis et al., 2008), focal segmental glomerular sclerosis (Huang et al., 2020), atherosclerosis (Niculescu et al., 1987), and lung fibrosis (Cipolla et al., 2017). Whether the C5b_Ct_-9 with increased lytic activity is involved in the development of tubal fibrosis requires further investigation. C6^−/−^ mice will be a powerful tool to address this question in future experiments.

The next key question was to identify which protein activated the novel cleavage pattern of C5 induced by *C. trachomatis* infection. To our knowledge, all C5 convertases are serine proteases (Rawal and Pangburn, 2001). The results of proteomic analysis narrowed the candidate C3-independent C5 convertases during the *C. trachomatis* infection to TPP, CPAF and cHtrA. Our results also demonstrated that the occurrence of C5 cleavage was consistent with the expression profile of active CPAF (Zhong et al., 2001; Huang et al., 2008), and EB that lack of CPAF (Zhong et al., 2001) failed to cleave C5. Penicillin treatment both inhibited the expression of CPAF and the cleavage of C5 induced by chlamydial infection. These observations enhanced the correlation between CPAF and C5 activation, and the subsequent finding that the *C. trachomatis* CPAF^−/−^ strain showed no C3-independent C5 activation confirmed this correlation and the causality, but not the direct interaction of CPAF and C5. Lactacystin, as a CPAF inhibitor reported previously (Zhong et al., 2001; Huang et al., 2008), failed to inhibit C5 cleavage induced by *C. trachomatis* in the current study, although there was an ambiguous inhibitory trend observed in the group of 1:2 dilution of lysates. However, this result did not confirm absence of an interaction between CPAF and C5 because an interaction between lactacystin and HeLa cells has previously been reported (Xu et al., 2015). The HeLa cells lysates in the co-incubation system in the current study may have predominantly consumed lactacystin, thus the concentration of lactacystin may be less effective in inhibiting CPAF. This inherent limitation of the current study means further investigation is required to determine whether there is a direct interaction between CPAF and C5.

It’s worth considering that CPAF, a chlamydial secreted serine protease, is a probable candidate as it has been demonstrated to have the potential ability to cleave or degrade a wide range of host proteins (Zhong, 2009), even though most cleavages and degradations were artifacts during the lysate preparation (Chen et al., 2012). Unlike the host proteins structurally isolated from CPAF in intact cells, complement components are widely distributed in body fluids, and the likelihood of CPAF encountering C5 is fairly high. It has been hypothesized that accumulation and storage of CPAF inside the inclusion and the cytosol of *C. trachomatis*-infected cells would be released with the EBs, the infectious form of *C. trachomatis*, into body fluid during the cell lysis process in the late development cycle. Moreover, CPAF has been proven to efficiently neutralize complement factor C3-dependent antichlamydial activity dependent on CPAF-mediated degradation of complement factor C3 and factor B (Yang et al., 2016). These findings led to the proposal of crosstalk between CPAF and complement system. We therefore hypothesized that CPAF might be the C3-independent C5 convertase during chlamydial infection. Nevertheless, our data did not entirely rule out the possibility that a host cell-derived factor induced by CPAF cleaves C5 directly. The direct interaction between CPAF and C5 needs to be fully confirmed by co-incubation of purified CPAF and C5. Regardless of whether CPAF directly cleaved C5, since the cleavage patterns of C5 mediated by thrombin and *C. trachomatis* infection are quite similar, they may have several common substrates. The key role of thrombin in the coagulation cascade (Amara et al., 2010) implies that there might be crosstalk between the coagulation system and *C. trachomatis* infection, and this theory requires further exploration.

NM, a synthetic serine protease inhibitor, has been approved by the Food and Drug Administration (FDA) for the treatment of acute pancreatitis and disseminated intravascular coagulation (Makino et al., 2016). Our previous study revealed that NM inhibited the growth of *Chlamydia*, but the underlying mechanisms remain unclear (Peng et al., 2020). Considering the good safety profile of NM, this serine protease inhibitor is expected to become a promising antichlamydial drug and be approved for clinical use driven by the drug repurposing strategy. Based on the ability of NM to inhibit a variety of serine proteases, chlamydial serine proteases CPAF and CtHtrA are obvious candidate targets. Therefore, in the current study, NM was used experimentally to regulate C5 activation triggered by *C. trachomatis* infection, and the results demonstrated a strong inhibitory effect of NM. A molecular docking showed NM bound to the active site of CPAF in a stable inhibitory conformation, which indicated the inhibition of NM on CPAF. Combining the inhibition of NM on C5 activation triggered by *C. trachomatis* infection, it partly strengthened the evidence that CPAF directly cleaved the C5. However, the inhibition of NM on CPAF cannot fully explain the efficacy of NM against *Chlamydia*. High doses of NM can eliminate the inclusion of *Chlamydia* in host cells (Peng et al., 2020), but CPAF is not an essential protein for *Chlamydia* to complete its development cycle, even though the CPAF^−/−^ strain has less infectious yield than the wild-type strain (Snavely et al., 2014). Therefore, further work is necessary to determine whether there is an interaction between NM and other chlamydial serine proteases, such as CtHtrA, an essential protein for *Chlamydia* during the development cycle.

In summary, a novel cleavage pattern of C5 induced by *C. trachomatis* infection *via* CPAF is proposed. C5 and C5b were modified as new products C5_Ct_ and C5b_Ct_ *via* cleavage at site K970, and C5b_Ct_ finally combined with the late components C6 to C9 to assemble C5b_Ct_-9 that exhibited more lytic activity than C5b-9. NM strongly inhibited C5 activation induced by chlamydial infection. These discoveries illuminate the mechanism of a C3-independent C5 activation induced by chlamydial infection, and furthermore provide a potential therapeutic target and drug for preventing tubal fibrosis caused by chlamydial infection.

## Data Availability Statement

The datasets presented in this study can be found in online repositories. The names of the repository/repositories and accession number(s) can be found in the article/**Supplementary Material**.

## Author Contributions

LP, JG, ZH, LC, SL, YZ, HX, XS, XF and YF performed the experiments. LP, JG, HZ, LT, FW and JC designed the studies. LP, JG, ZH and LC performed statistical analysis. LP, JG and JC prepared the first draft of the manuscript. All authors contributed to the article and approved the submitted version.

## Funding

This study was supported by the National Natural Science Foundation of China (grant no. 31670178) and the Natural Science Foundation of Hunan Province, China (grant no. 2021JJ30918).

## Conflict of Interest

The authors declare that the research was conducted in the absence of any commercial or financial relationships that could be construed as a potential conflict of interest.

## Publisher’s Note

All claims expressed in this article are solely those of the authors and do not necessarily represent those of their affiliated organizations, or those of the publisher, the editors and the reviewers. Any product that may be evaluated in this article, or claim that may be made by its manufacturer, is not guaranteed or endorsed by the publisher.
